# Niche-localized tumor cells are protected from HER2-targeted therapy via upregulation of an anti-apoptotic program in vivo

**DOI:** 10.1038/s41523-017-0020-z

**Published:** 2017-05-01

**Authors:** Jason J. Zoeller, Roderick T. Bronson, Laura M. Selfors, Gordon B. Mills, Joan S. Brugge

**Affiliations:** 1 Department of Cell Biology, Harvard Medical School, 240 Longwood Avenue, Boston, 02115 MA USA; 2 Rodent Histopathology Core, Harvard Medical School, Boston, MA USA; 3 Systems Biology, UT MD Anderson Cancer Center, Houston, TX USA

## Abstract

Several lines of evidence suggest that components of the tumor microenvironment, specifically basement membrane and extracellular matrix proteins, influence drug sensitivities. We previously reported differential drug sensitivity of tumor cells localized adjacent to laminin-rich extracellular matrix in three-dimensional tumor spheroid cultures. To evaluate whether differential intra-tumor responses to targeted therapy occur in vivo, we examined the sensitivity of human epidermal growth factor receptor 2-positive tumors to lapatinib using a previously described ductal carcinoma in situ-like model characterized by tumor cell confinement within ductal structures surrounded by an organized basement membrane. Here we show that tumor cells localized to a ‘niche’ in the outer layer of the intraductal tumors adjacent to myoepithelial cells and basement membrane are resistant to lapatinib. We found that the pro-survival protein BCL2 is selectively induced in the niche-protected tumor cells following lapatinib treatment, and combined inhibition of HER2 and BCL-2/XL enhanced targeting of these residual tumor cells. Elimination of the niche-protected tumor cells was achieved with the HER2 antibody–drug conjugate T-DM1, which delivers a chemotherapeutic payload. Thus, these studies provide evidence that subpopulations of tumor cells within specific microenvironmental niches can adapt to inhibition of critical oncogenic pathways, and furthermore reveal effective strategies to eliminate these resistant subpopulations.

## Drug resistance: Overcoming localized resistance to anti-HER2 therapy

Location-specific subpopulations of breast cancer cells adapt to targeted drug treatment, but therapeutic strategies exist to attack these niche-protected cells. A team led by Joan Brugge and Jason Zoeller from Harvard Medical School, USA, implanted human HER2+ breast tumor cells into the ducts of mouse mammary glands to recapitulate the architecture of ductal carcinoma in situ, a common type of non-invasive breast cancer. They found that cancer cells located on the outer rim of the tumors were resistant to lapatinib, a drug that targets the HER2 protein. Combination treatment with lapatinib and a drug that blocks a pro-survival protein called BCL2 that was specifically enriched in the outer cells after lapatinib treatment helped kill more cells. Complete elimination of the resistant cells was achieved with an antibody-drug conjugate, T-DM1, that binds to HER2 and then releases a chemotherapeutic payload.

## Introduction

Extracellular matrix (ECM) proteins produced by diverse tumor types protect tumor cells from death in response to various agents.^3–6^ Work from our laboratory and others in three-dimensional (3D) culture systems has defined a protective role for ECM within the context of both normal^7^ and tumor^1^ cells. Using epithelial tumor cell lines cultured in reconstituted basement membrane (BM), we previously found that the outer, ECM-attached cells are resistant to multiple different drug therapies.^1^ ECM protection involved activation of an adaptive response program, including FOXO-dependent transcriptional and cap-independent translational induction of multiple receptor tyrosine kinases (RTKs) and pro-survival BCL2 family proteins.

To address whether a similar differential adaptive response is observed in vivo, we examined a tumor model that recapitulates the ECM-enveloped architecture of 3D spheroids by generating ductal carcinoma in situ (DCIS)-like tumors via intraductal injection of HER2+ SUM225 breast tumor cells.^2^ Since HER2+ DCIS accounts for 40–60% of all patient-related DCIS cases,^8–13^ this model represents one of the most relevant approaches to understand the biology of HER2+ DCIS and to evaluate HER2-targeted therapies within the context of pre-neoplastic breast cancer.

## Results

**Fig. 1:**
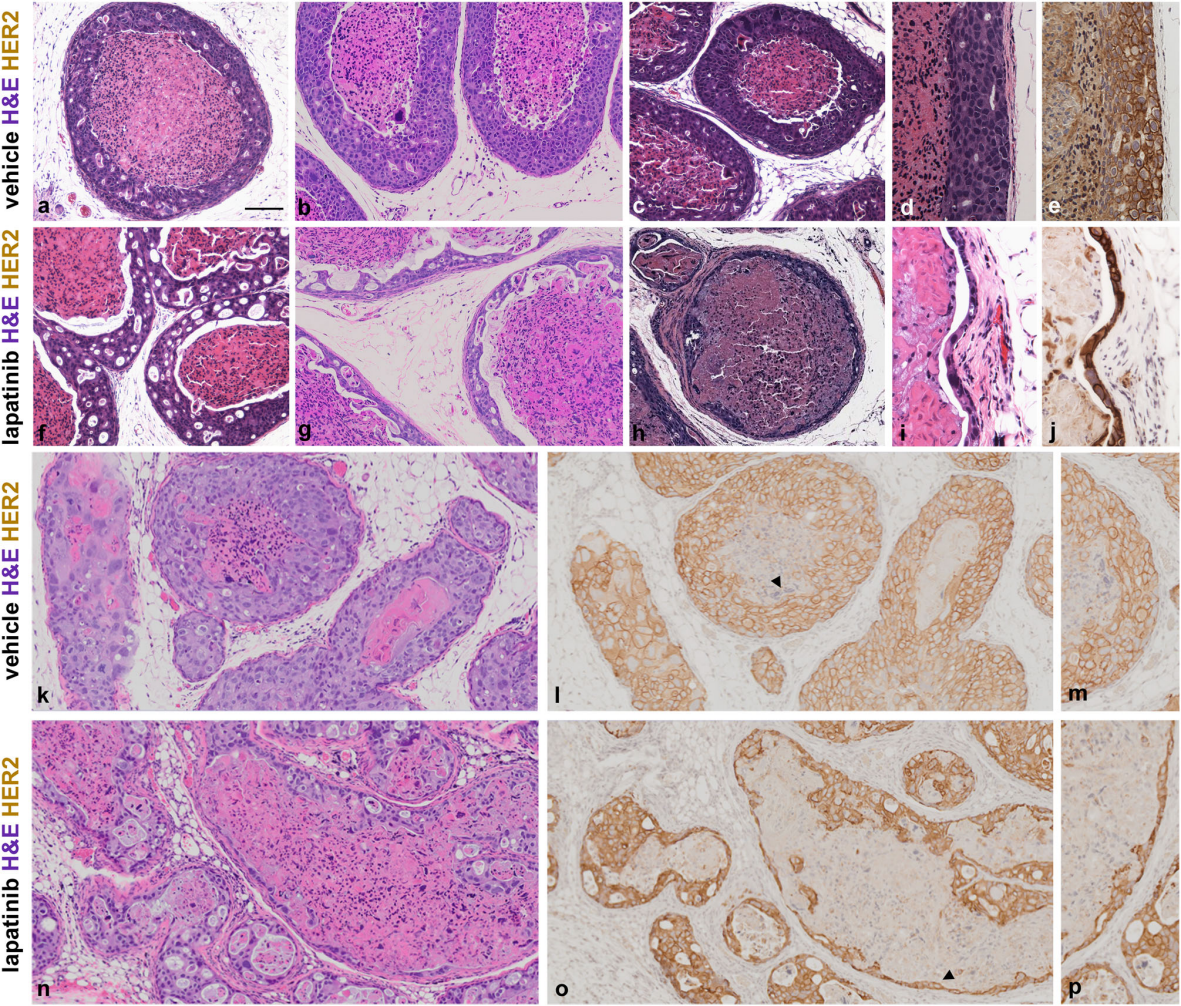
Preservation of niche-localized tumor cells post-lapatinib treatment. Representative H&E images of vehicle-treated (**a**–**e**) and 5-day or 10-day lapatinib-treated (**f**–**j**) SUM225 DCIS-like tumors. Note **f**, **g**, **i** were 10-day whereas **h** was 5-day lapatinib-treated. The lapatinib-treated tumor images represent the spectrum of responses observed, with the least significant reductions in the viable tumor cell content shown in (**f**). Note cell crypts (vacuole-like spaces throughout the cell layer) associated with the areas of cell death. More significant reduction in the viable tumor cell content is shown in (**g**, **h**), with maintenance of only the outermost layer of tumor cells adjacent to myoepithelial cells and BM in (**h**). HER2 immunostains confirm HER2 status among the residual tumor cell population (**j**). H&E (**d**, **i**) and serial section HER2 IHC (**e**, **j**) are presented. Note **d**, **e**, **i**, **j** represent longitudinal sections, whereas all other panels represent transverse sections, through the intraductal tumors. Comparison of vehicle (**k**–**m**) and 21-day lapatinib-treated (**n**–**p**) SUM225 tumors. Representative H&E (**k**, **n**) and serial section HER2 IHC (**l**, **m**) and (**o**, **p**) are presented. *Arrowheads* in **l**, **o** highlight the regions in **m**, **p**. Note preservation of the niche-localized HER2+ tumor cells post-long term lapatinib treatment (**p**). Scale bar, ~200 μm

To generate intraductal DCIS-like tumors, SUM225 breast tumor cells were transplanted via cleaved nipple injection into the mammary gland of 6–10-week-old female NOD/scid mice. The intraductal tumors recapitulated the histological architecture of human DCIS,^2,14^ with multiple layers of human epidermal growth factor receptor 2-positive (HER2+) tumor cells confined within a laminin-rich BM and a centralized necrotic core (Supplementary Fig. 1). SUM225 cells are resistant to trastuzumab, a HER2-targeted monoclonal antibody, but are sensitive to lapatinib, a small molecule dual RTK inhibitor of HER2 and epidermal growth factor receptor (EGFR).^15–17^ To examine the differential drug sensitivity of spatially distinct tumor cells in this model, female NOD/scid mice bearing HER2+ SUM225 DCIS-like tumors were randomized into two treatment groups: lapatinib monotherapy (200 mg/kg/day) or vehicle alone for a period of 5–10 days (*n* = 4–5 mice per group). Endpoint comparison of tumor sections by hematoxylin and eosin (H&E) analysis revealed that lapatinib treatment induced a reduction in the number of tumor cell layers, but did not eliminate the tumor cells closest to the BM (Fig. 1). The extent of tumor cell death and reduction of tumor mass varied in different regions of the tumor and in different experiments; however, under conditions in which a near complete reduction of the tumor cell layers was observed, the outer layer of tumor cells in contact with the myoepithelial cells and BM was spared (Fig. 1). HER2 IHC identified residual HER2+ tumor cells and indicated maintenance of HER2 status post-lapatinib (Fig. 1). Lapatinib treatment for a longer period, 21 days, resulted in similar preservation of these tumor cell populations (Fig. 1). Ki67 IHC revealed an overall reduction in the proliferation positive tumor cell population post-lapatinib (Supplementary Fig. 2). Interestingly, a subset of outer layer tumor cells maintain proliferative capacity post-treatment (Supplementary Fig. 2).

We also examined another HER2+ tumor cell line, SUM190,^18^ that can generate intraductal tumors after intraductal transplantation. SUM190 maintain a non-invasive phenotype in vivo with histological similarities to SUM225. However, this model was uninformative with respect to differential drug sensitivity because both the outer and niche-associated tumor cells were insensitive to lapatinib, possibly due to a H1047R *PIK3CA* mutation, which has been shown to limit the effectiveness of lapatinib^19^ (Supplementary Fig. 3).

**Fig. 2:**
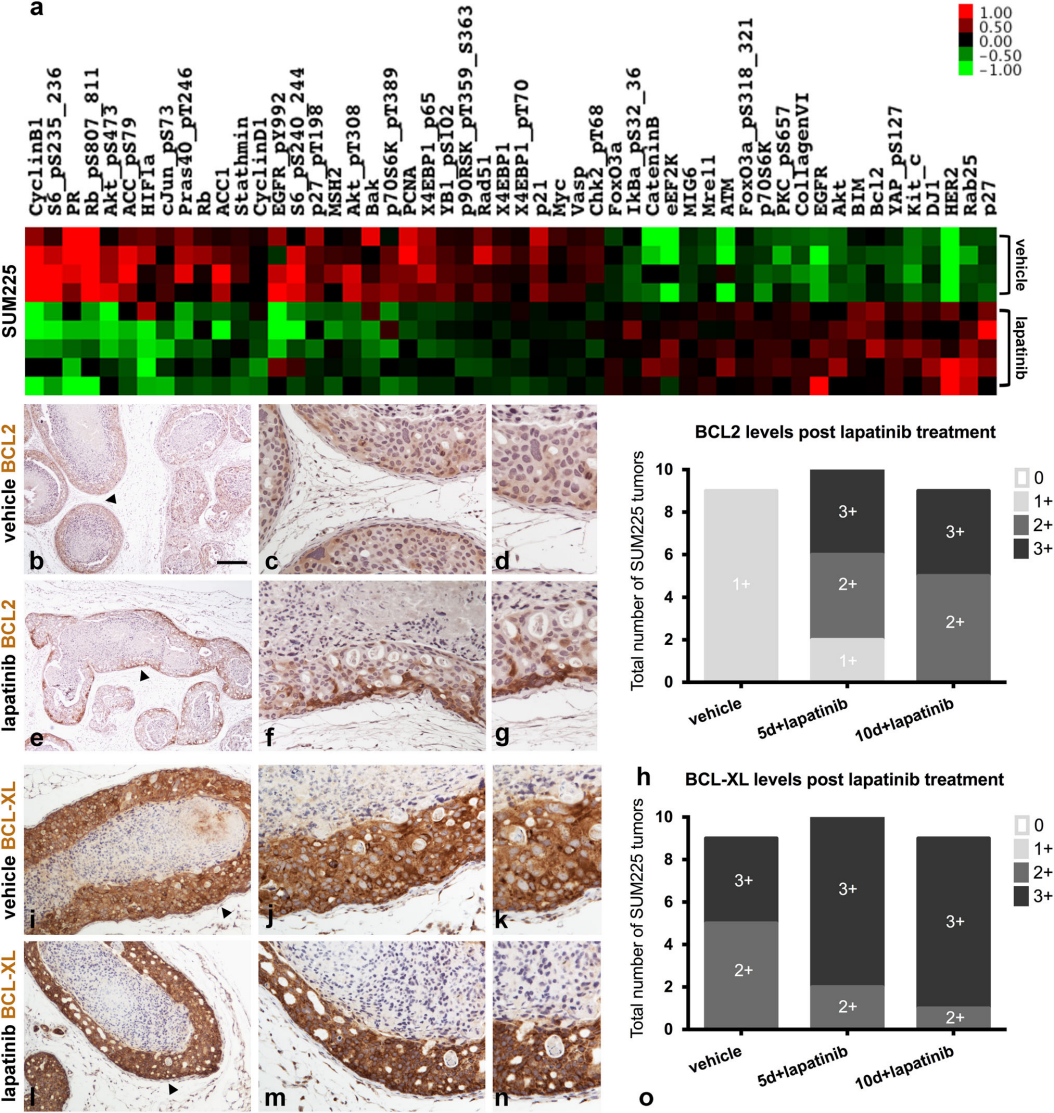
Lapatinib induces pro-survival BCL2 in the niche-protected tumor cells. **a** RPPA analysis of SUM225 vehicle and 10-day lapatinib-treated tumors. Proteins with significant differences (unpaired two-tail Welch’s *t* test *p* value < 0.05) between lapatinib-treated and vehicle-treated tumors are shown. Data are log2 transformed and median centered. Statistical analysis was performed in R v.3.2.2. The RPPA heatmap was generated in Cluster v.3.0 and Java TreeView v.1.1.1. **b**–**g** Matched tumor sections were assayed for BCL2 via IHC. Representative vehicle-treated and lapatinib-treated tumors from two independent experiments are presented. IHC assays confirmed the RPPA results and highlighted selective BCL2 induction within niche-localized tumor cells. **h** BCL2 levels were scored according to intensity as 0, 1+, 2+, or 3+ and summarized across multiple tumors from two independent experiments (Fisher’s exact test vehicle vs. 5-day lapatinib; *p* value = 0.0007145 and vehicle vs. 10-day lapatinib *p* value = 4.114e-05). Post lapatinib, BCL2 expression was largely localized within the outermost tumor cells. **i**–**o** Lapatinib treatment did not alter expression of the pro-survival protein BCL-XL. Note SUM225 tumors express moderate to high BCL-XL levels with or without treatment and throughout all viable cells (Fisher’s exact test vehicle vs. 5-day lapatinib; *p* value = 1.083e-05 and vehicle vs. 10-day lapatinib *p* value = 4.114e-05). Fisher’s exact tests were performed in R v.3.2.2. *Arrowheads* (**b**, **e**, **i**, **l**) highlight regions magnified in subsequent panels. Scale bar, ~200 μm

To explore potential mechanisms underlying the adaptation of SUM225 tumors to lapatinib treatment, we performed reverse phase protein arrays (RPPAs)^20^ on protein extracts prepared from vehicle-treated (*n* = 4) or lapatinib-treated (*n* = 5) tumor fragments (Fig. 2a). RPPA profile analysis confirmed that pathways downstream of HER2 and EGFR were inhibited post treatment (e.g., phosphorylation of EGFR, AKT and its targets, and mTOR targets). However, multiple RTKs, including HER2 and EGFR, were elevated in response to lapatinib treatment. Both observations are consistent with a previously described adaptive response mechanism mediated by release of feedback inhibition of pathways regulated by lapatinib^1,21,22^ and/or lapatinib-mediated stabilization and accumulation of HER2 protein.^23–25^

RPPA analysis also revealed an induction of BCL2 following lapatinib treatment (Fig. 2). This result is consistent with our previous in vitro data identifying BCL2 as a component of the adaptive response to PI3K/mTOR inhibition in ovarian cancer cells.^1^ To determine whether BCL2 was induced throughout the entire tumor or specifically enriched in the protected outer cells, we performed BCL2 IHC analysis on matched tumor tissue sections (Fig. 2). Blinded pathological assessment confirmed BCL2 induction following lapatinib treatment, and revealed that it selectively occurred in the outermost, lapatinib-resistant cells of the intraductal tumors (Fig. 2 and Supplementary Fig. 4). BCL2 induction was not coupled to estrogen receptor expression,^26^ as SUM225 tumors maintained an ER-negative phenotype (Supplementary Fig. 5).^27^ In contrast to BCL2, BCL-XL did not exhibit a similar BM-restricted pattern nor a notable change in expression level in response to lapatinib treatment (Fig. 2). The levels of BCL-XL were elevated throughout the entire tumor in lapatinib-treated tumors (Fig. 2).

**Fig. 3:**
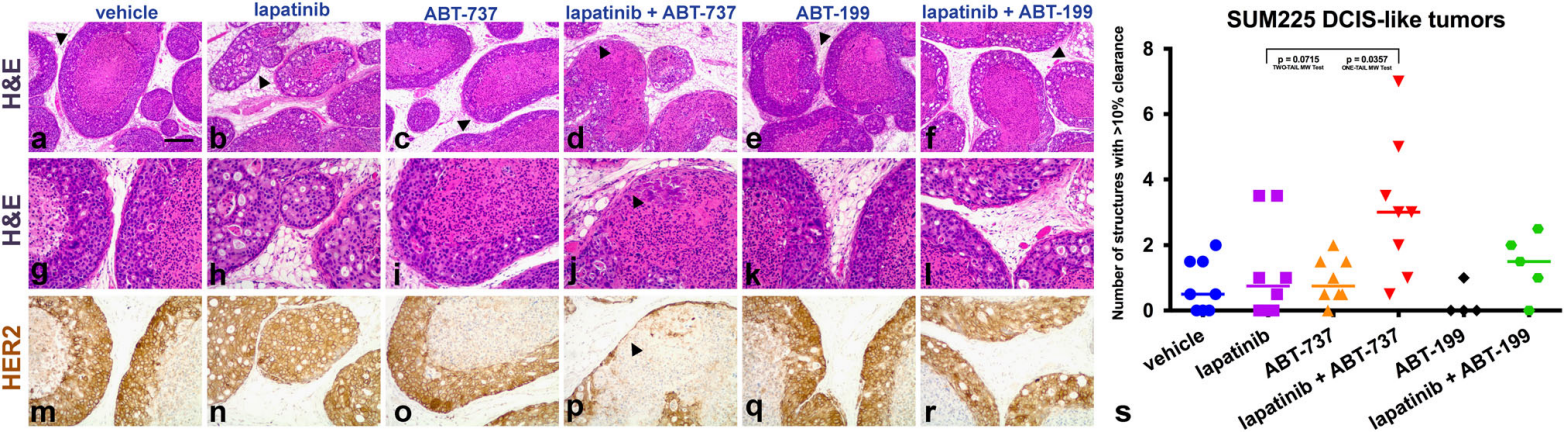
Inhibition of BCL-2/XL in combination with lapatinib treatment eliminates a subset of the niche-localized tumor cells. Representative H&E low magnification (**a**–**f**) and high magnification (**g**–**l**) images are presented to compare treatment groups. Serial sections were subjected to HER2 IHC in order to visualize tumor cells. The matched HER2 stains are presented alongside the H&E stained sections (**m**–**r**). *Arrowheads* (**a**–**f**) highlight regions presented in **g**-**l**. Note multiple viable HER2+ tumor cells (**g**–**i**, **k**). Note complete elimination of HER2+ tumor cells (**j**, *arrowhead*) in response to lapatinib plus ABT-737 (dual BCL-2/XL inhibitor). Lapatinib plus ABT-199 (BCL2-selective inhibitor) treatment did not cause elimination of niche-localized cell populations (**l**). Graph (**s**) shows the percent loss of the outer niche-localized cells (clearance, quantified as described in the Methods) in multiple tumors from two independent experiments (Mann–Whitney test lapatinib vs. lapatinib + ABT-737; *p* value = 0.0357 one-tail and *p* value = 0.0715 two-tail). Each line represents the median. Scale bar, ~200 μm

To examine the functional relevance of BCL2 induction in the BM-localized tumor cells, we examined the effects of lapatinib treatment in combination with the dual BCL-2/XL inhibitor ABT-737.^28^ Mice bearing SUM225 DCIS-like tumors (*n* = 4–5 mice per group) were pre-treated with either vehicle or lapatinib [200 mg/kg/day per os (p.o.)] for 10 days followed by 5 days of either vehicle or lapatinib plus ABT-737 [70 mg/kg/day intraperitoneally (i.p.)]. Analyses of the tissue sections by H&E in parallel with HER2 IHC indicated that tumors treated with ABT-737 alone were indistinguishable from matched vehicle-treated tumors (Fig. 3). The combination treatment resulted in either greater reduction of the tumor cell layer or complete elimination of the residual tumor cells, including the outer layer of cells proximal to the basement membrane (Fig. 3). Quantification of these features revealed an overall increase in the loss of cells at the BM zone after treatment with lapatinib (Fig. 3s). ABT-737 treatment induced thrombocytopenia, a marker of ABT-737 pharmacodynamics in vivo, confirming on-target action in these experiments (Supplementary Fig. 6).^29^ Although the 5-day combination treatment did not eradicate the entire tumor, the enhancement in cell death indicates that BCL2 family proteins contribute to the adaptive survival response to lapatinib. Longer-term combination treatment was not feasible due to weight loss exceeding AAALAC limits (Supplementary Fig. 6).

To address whether selective inhibition of BCL2 in combination with HER2 blockade would induce a similar response, we examined the consequences of lapatinib treatment in combination with the BCL2-specific inhibitor ABT-199.^29^ ABT-199 treatment did not induce thrombocytopenia, which was consistent with BCL2-specific inhibition^29^ (Supplementary Fig. 6). Notably, the combination of lapatinib and ABT-199 did not significantly enhance the effects of lapatinib monotherapy (Fig. 3), highlighting the importance of ABT-737-induced BCL-XL inhibition as a critical aspect of combination treatment effectiveness. This result is consistent with our observation of constitutive BCL-XL expression within all cells of the tumor (Fig. 2).

**Fig. 4:**
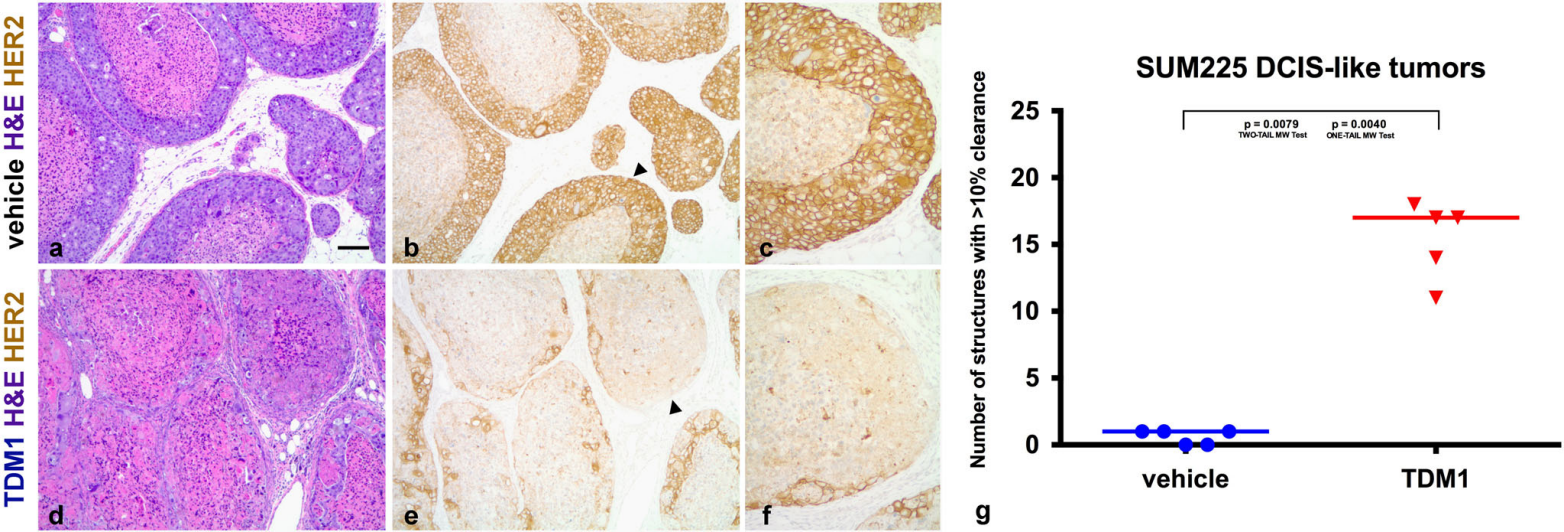
T-DM1 treatment eliminates the outer and inner tumor cells. Representative H&E stained sections from vehicle-treated (**a**) and T-DM1-treated (**d**) SUM225 tumors are presented alongside serial section HER2 IHC (**b**, **c**) and (**e**, **f**). *Arrowheads* in **b**, **e** highlight the tumor nests that are presented in **c**, **f**. Note the significant elimination of HER2+ tumor cells post-T-DM1 treatment (**e**, **f**). Note niche-localized, and non-localized, cells are ultrasensitive to T-DM1 treatment. Pathological assessment of clearance was performed as described in the methods (**g**). Data was summarized across five vehicle-treated and five T-DM1-treated SUM225 tumors (Mann–Whitney test vehicle vs. T-DM1; *p* value = 0.0040 one-tail and *p* value = 0.0079 two-tail). Each *line* represents the median. Scale bar, ~100 μm

We also examined whether chemotherapeutic agents would provide better targeting and elimination of the protected tumor cell populations. As a means to deliver a cytotoxic agent directly to the HER2+ tumor cells, we utilized the HER2 ADC T-DM1. T-DM1 combines the anti-HER2 properties of trastuzumab with the anti-microtubule cytotoxic activities of DM1.^30–32^ Mice bearing SUM225 DCIS-like tumors were randomized into one of two treatment groups (*n* = 5 mice per group): single agent T-DM1 (10 mg/kg/1 × week i.p. 2 weeks) or vehicle. Analyses of the tissue sections by H&E in parallel with HER2 IHC indicated that T-DM1 treatment induced extensive cell death within the inner tumor cell layers as well as large sections of the outer, protected tumor cell population (Fig. 4). Quantification of these features revealed an overall enrichment of cell death at the BM zone (Fig. 4g). These results highlight T-DM1 as a means to eliminate niche-protected tumor cells.

## Discussion

Here we identified a DCIS sub-population of niche-associated tumor cells, adjacent to myoepithelial cells and BM, that are resistant to lapatinib and induce BCL2 post treatment. We found that HER2 blockade in combination with a BCL-2/XL inhibitor reduced the niche-protected population. However, the most effective elimination of these cells was observed after treatment with the antibody-drug conjugate, T-DM1, which delivers cytotoxic DM1 to HER2+ tumor cells.

While the precise mechanism responsible for lapatinib resistance within niche-localized tumor cells isn’t known, we predict that the BM, synthesized and organized by the myoepithelial cells, may be the most critical component of the protective response. Our previous studies, which utilized 3D tumor spheroids in vitro cultured in the absence of myoepithelial cells or other cellular tumor microenvironment components, revealed a similar differential resistance of the outer, matrix-attached cells treated with PI3K and/or mTOR inhibitors.^1^ In the in vivo DCIS-like model presented here, BCL2 upregulation was clearly enriched specifically in the outer, niche-localized drug resistant tumor cell populations. The lack of BCL2 upregulation in the remainder of the tumor suggests that cells beyond the ‘niche’ lack the abilities to adapt in a similar manner.

Outside of HER2+ disease, BCL-2/XL inhibition in combination with tamoxifen has proven efficacy in patient-derived xenograft (PDX) models of ER+ breast cancer.^33^ BCL2 is a known ER responsive gene, rendering it an especially attractive target for ER+ breast tumors,^34^ which exhibit a strong correlation with BCL2 expression regardless of HER2 status.^35–37^ BCL-2/XL blockade in combination with docetaxel also has proven efficacy in PDX models of triple negative breast cancer.^38^ Unlike SUM225 tumor cells treated with lapatinib, niche-localized SUM225 tumor cells were sensitive to a chemotherapeutic agent. These results highlight T-DM1 treatment as a means to prevent the persistence of niche-localized drug resistant tumor cells in vivo.

The adaptive response demonstrated here could facilitate the acquisition of additional genetic alterations in proliferating tumor cells that may lead to the generation of stably resistant tumor cells that can survive and proliferate in an niche-independent fashion. This could represent one mechanism for tumor recurrence in patients treated with targeted therapies. Indeed, two independently generated tumor cell models of acquired lapatinib resistance have identified BCL2 overexpression as a component of the resistant phenotype^26,39^ and suggest that BCL2 upregulation via the adaptive response (de novo resistance) could be maintained by stable, inherited alterations that lead to long-term acquired resistance. In line with this evidence, a recent report from a substantially large patient cohort supports reclassification of patients according to pCR or pCR + DCIS in correlation with disease-free survival.^40^

Our results highlight the potential for similar niches to confer protection in other tumor types. Since the BM is a notable component of prostate,^41^ lung^3,42^ and skin,^43^ tumor cells adjacent to BM could also be protected from targeted-therapies in this context. Indeed, in a mouse prostate non-invasive tumor model induced by PTEN deletion, outer tumor cells only were preserved in mice treated with mTOR inhibitors and residual tumor cells were BCL2-positive.^44^ The extent to which these protected niches are relevant to human tumors treated with targeted-therapies warrants future investigation. Although the relevant niche-associated components that confer protection in vivo remain to be defined, our results underscore the importance of microscopic evaluation of individual tumor cell responses to therapeutic agents in order to reveal spatially restricted heterogeneous drug responses. The identification and characterization of intra-tumoral protective microenvironmental niches may guide targeted therapeutic strategies to prevent drug resistance and disease recurrence in the future.

## Materials and methods

### Cell culture

HER2+ SUM225 and SUM190 breast tumor cells^18,45^ were maintained in complete media (Ham’s F12 supplemented with 5% heat-inactivated fetal bovine serum, 1 µg/ml hydrocortisone, 5 µg/ml insulin, 1% penicillin-streptomycin, 10 mM HEPES) by standard tissue culture practice at 37 °C and 5% CO_2_. SUM225 were kindly provided by Fariba Behbod (KU Medical Center). SUM190 were kindly provided by Dennis Slamon (UCLA).

### Intraductal human-in-mouse tumor cell transplantation

Intraductal tumor cell transplants were performed as previously described.^2^ SUM225 or SUM190 cells were washed twice in PBS, trypsinized, counted and resuspended in complete media plus 0.1% trypan blue. Briefly, ~30 × 10^3^ tumor cells in 3–5 µl were injected via cleaved nipple into the mammary gland of 6–10-week-old female NOD/scid mice. A 5 µl Hamilton syringe (75rn, G32, 2”, 90°) was used for all transplants. All animal procedures were carried out according to IACUC04004 and Harvard ARCM policy.

### Tumor histology and IHC

Tumor tissue was harvested at the experimental endpoint and fixed in 3.7% paraformaldehyde (Boston BioProducts) or 10% neutral buffered formalin (Sigma). Tissue was processed for paraffin embedding, sectioning and H&E staining by the Harvard Rodent Histopathology core. Unstained sections were analyzed by immunohistochemical analysis according to standard protocol. For expanded details on immunohistochemistry methods and antibodies, please refer to the Supplementary Methods.

### Drug treatment in vivo

For in vivo studies, lapatinib^15,46–48^ (GlaxoSmithKline) was administered p.o. at 200 mg/kg once per day. Lapatinib was prepared according to GlaxoSmithKline recommendations as a suspension in 0.5% hydroxypropylmethylcellulose (Dow Chemical K-15M) plus 0.1% Tween 80 (Sigma) in water. ABT-737^28^ (AbbVie) was administered i.p. at 70 mg/kg once per day. ABT-737 was prepared according to AbbVie recommendations in 30% propylene glycol (Sigma) plus 0.5% Tween 80 (Sigma) and 65% D5W (5% dextrose in water, Sigma) pH 3-4. ABT-199^29^ (AbbVie) was administered p.o. at 70 mg/kg once per day. ABT-199 was prepared according to AbbVie recommendations in 60% PHOSAL 50 PG (Lipoid) plus 30% polyethylene glycol 400 (Dow Chemical) and 10% ethanol. T-DM1^30^ (Genentech) was administered i.p. at 10 mg/kg once per week. T-DM1 was prepared according to Genentech recommendations in sterile water for injection (GIBCO). Matched animals received vehicle alone in the same manner as drug-treated counterparts. All animals were weighed and randomized into groups before treatment. Either the average weight (average of the largest and smallest animals) or individual weights were used for dose calculations. Lapatinib treatment experiments were performed with or without a short-term food fast period. Mice were deprived of food for 2 h before the treatment and for 1 h after the treatment. Retro-orbital blood collection was performed on ABT-treated and vehicle-treated mice at the experimental endpoint. Mouse platelet counts were measured by Charles River Research Animal Diagnostic Services (Wilmington, MA).

### Reverse phase protein arrays

Fresh tumor tissue was snap frozen in liquid nitrogen and stored at −80 °C. For protein lysates, the tumor fragments were pulverized over liquid nitrogen and resuspended in RPPA lysis buffer or were sonicated in RPPA lysis buffer (1% Triton X-100, 50 mM HEPES pH 7.4, 150 mM NaCl, 1.5 mM MgCl_2_, 1 mM ethylene glycol-bis(β-aminoethyl ether)-N,N,N',N'-tetraacetic acid, 100 mM NaF, 10 mM NaPPi, 10% glycerol, 1 mM Na_3_VO_4_ plus 1 mM phenylmethylsulfonyl fluoride and 10 μg/ml aprotinin or protease (Roche 11697498001) and phosphatase (Roche 04906837001) inhibitor tablets). Lysates were mixed well and clarified by centrifugation at 13000 rpm for 10 min at 4 °C. Supernatants were collected and protein was quantified by BCA assay (Pierce). Cellular proteins were denatured by 1% sodium dodecyl sulfate (SDS) (with β-mercaptoethanol) and diluted in five twofold serial dilutions in dilution buffer (lysis buffer containing 1% SDS). Serial diluted lysates were arrayed on nitrocellulose-coated slides (Grace Biolab) by an Aushon 2470 Arrayer (Aushon BioSystems). A total of 5808 array spots were arranged on each slide including the spots corresponding to positive and negative controls prepared from mixed cell lysates or dilution buffer, respectively. Each slide was probed with a validated primary antibody plus a biotin-conjugated secondary antibody. Only antibodies with a Pearson correlation coefficient between RPPA and Western blotting of greater than 0.7 were used in reverse phase protein array studies. Antibodies with a single or dominant band on Western blotting were further assessed by direct comparison to RPPA using cell lines with differential protein expression or modulated with ligands, inhibitors or siRNA for phospho-proteins or structural proteins, respectively. The signal obtained was amplified using a Dako Cytomation-catalyzed system (Dako) and visualized by 3,3'-diaminobenzidine colorimetric reaction. The slides were scanned, analyzed, and quantified using Array-Pro Analyzer software (MediaCybernetics) to generate spot intensity. Each dilution curve was fitted with a logistic model (“Supercurve Fitting” developed by the Department of Bioinformatics and Computational Biology, MD Anderson Cancer Center). The model fits a single curve using all the samples (i.e., dilution series) on a slide with the signal intensity as the response variable, and the dilution steps as the independent variable. The fitted curve is plotted with the signal intensities, both observed and fitted, on the *y*-axis and the log2-concentration of proteins on the *x*-axis for diagnostic purposes. The protein concentrations of each set of slides were then normalized by median polish, which was corrected across samples by the linear expression values using the median expression levels of all antibody experiments to calculate a loading correction factor for each sample.

### Microscopy

H&E and IHC images were captured on the laboratory Nikon Eclipse E200 microscope equipped with an Idea color camera and the SPOT software package. H&E and IHC images were also captured, at the Nikon Imaging Center at Harvard Medical School, on the Nikon 80i upright microscope equipped with a PriorProScanII motorized stage and a Nikon Digital Slight DS Fi1 color camera. The NIS-Elements software package was used for image acquisition and analysis. H&E and IHC images were scanned, at the Neurobiology Imaging Facility at Harvard Medical School, on the Olympus VS120-S5 microscope equipped with a Hamamatsu ORCA-R2 color camera. The VS-ASW-FL software package was used for image analysis.

### Quantification of clearance

Pathological assessment of clearance was performed by rodent pathologist Dr. Roderick Bronson, who was blinded to tumor treatment identities. On average, 30 DCIS-like structures were evaluated from multiple tumors per treatment group (Supplementary Tables 1 and 2). DCIS-like structures were scored according to the percent of niche-localized cells eliminated per structure (clearance). The total number of structures with greater than 10% clearance was graphed per tumor in Figs. 3 and 4.

### Quantification of Ki67

Pathological assessment of Ki67 was performed by rodent pathologist Dr. Roderick Bronson, who was blinded to tumor treatment identities. Twenty-five 10× fields were evaluated from multiple vehicle-treated and lapatinib-treated tumors. Fields were scored according to the percent of Ki67+ cells (numerical values 0–100) and to the localization of Ki67+ cells (categorical values outer Ki67 positive or negative). The percent average Ki67+ cells per 10× field was graphed per tumor in Supplementary Fig. 2g. The percent of outer Ki67+ cells per 10× field was graphed per tumor in Supplementary Fig. 2h.

### Statistics

Statistical analyses (except where noted) were performed using GraphPad Prism version 6 for MAC.

## Electronic supplementary material

Supplementary Items

Supplementary Figure 1

Supplementary Figure 2

Supplementary Figure 3

Supplementary Figure 4

Supplementary Figure 5

Supplementary Figure 6
