# Erratum: Niche-localized tumor cells are protected from HER2-targeted therapy via upregulation of an anti-apoptotic program in vivo

**DOI:** 10.1038/s41523-017-0031-9

**Published:** 2017-09-22

**Authors:** Jason J. Zoeller, Roderick T. Bronson, Laura M. Selfors, Gordon B. Mills, Joan S. Brugge

**Affiliations:** 1 Department of Cell Biology, Harvard Medical School, 240 Longwood Avenue, Boston, MA 02115 USA; 2 Rodent Histopathology Core, Harvard Medical School, Boston, MA USA; 3 Systems Biology, UT MD Anderson Cancer Center, Houston, TX USA

**Erratum to:** *npj Breast Cancer* (2017); doi:10.1038/s41523-017-0020-z; Published 01 May 2017

Two references were mistakenly omitted from the first sentence of the introduction.

Original text:

Extracellular matrix (ECM) proteins produced by diverse tumor types protect tumor cells from death in response to various agents.^1,2,3,4^

1. Sethi, T. *et al*. Extracellular matrix proteins protect small cell lung cancer cells against apoptosis: a mechanism for small cell lung cancer growth and drug resistance in vivo. *Nat. Med*. **5**, 662–668 (1999).

2. Sherman-Baust, C. A. *et al*. Remodeling of the extracellular matrix through overexpression of collagen VI contributes to cisplatin resistance in ovarian cancer cells. *Cancer Cell* **3**, 377–386 (2003).

3. Uhm, J. H., Dooley, N. P., Kyritsis, A. P., Rao, J. S. & Gladson, C. L. Vitronectin, a glioma-derived extracellular matrix protein, protects tumor cells from apoptotic death. *Clin. Cancer Res*. **5**, 1587–1594 (1999).

4. Pupa, S. M. *et al*. Regulation of breast cancer response to chemotherapy by fibulin-1. *Cancer Res*. **67**, 4271–4277 (2007).

Corrected text:

Extracellular matrix (ECM) proteins produced by diverse tumor types protect tumor cells from death in response to various agents.^1,2,3,4,5,6^

1. Muranen, T. *et al*. Inhibition of PI3K/mTOR leads to adaptive resistance in matrix-attached cancer cells. *Cancer Cell* **21**, 227–239 (2012).

2. Behbod, F. *et al*. An intraductal human-in-mouse transplantation model mimics the subtypes of ductal carcinoma in situ. *Breast Cancer Res*.  **11**, R66 (2009).

3. Sethi, T. *et al*. Extracellular matrix proteins protect small cell lung cancer cells against apoptosis: a mechanism for small cell lung cancer growth and drug resistance in vivo. *Nat. Med*. **5**, 662–668 (1999).

4. Sherman-Baust, C. A. *et al*. Remodeling of the extracellular matrix through overexpression of collagen VI contributes to cisplatin resistance in ovarian cancer cells. *Cancer Cell* **3**, 377–386 (2003).

5. Uhm, J. H., Dooley, N. P., Kyritsis, A. P., Rao, J. S. & Gladson, C. L. Vitronectin, a glioma-derived extracellular matrix protein, protects tumor cells from apoptotic death. *Clin. Cancer Res*. **5**, 1587–1594 (1999).

6. Pupa, S. M. *et al*. Regulation of breast cancer response to chemotherapy by fibulin-1. *Cancer Res*. **67**, 4271–4277 (2007).

All subsequent references have been renumbered accordingly.

These errors have now been corrected in the HTML and PDF versions of this article.

