# Supplementary material for: Niche-localized tumor cells are protected from HER2-targeted therapy via upregulation of an anti-apoptotic program in vivo
Source: NPJ Breast Cancer. 2017 May 1;3:18. doi: 10.1038/s41523-017-0020-z (PMC5460247; doi:10.1038/s41523-017-0020-z)
Supplement: Supplementary file 1 — Supplementary Items [file 41523_2017_20_MOESM1_ESM.docx]

**Supplementary Figure Legends**

**Supplementary Figure 1.** SUM225 intraductal (DCIS-like) tumors. Intraductal transplantation of SUM225 tumor cells results in the establishment of DCIS-like tumor xenografts. H&E cross-sections through the mouse mammary gland (**a**) indicate non-invasive DCIS-like tumor formation characterized by central comedo necrosis. HER2 IHC (**b**) on serial sections labels the HER2+ SUM225 tumor cells. Note the HER2+ tumor cells (**c** and **d**) are confined within the boundaries of a laminin-rich basement membrane (**e**) and myoepithelial cell layer (**f**). Scale bar, ~100 μm.

**Supplementary Figure 2.** SUM225 tumors exhibit proliferative blockade post-lapatinib. Comparison of vehicle- (**a-c**) and lapatinib-treated (**d-f**) SUM225 tumors. Representative H&E (**a** and **d**) and serial section Ki67 IHC (**b,c** and **e,f**) are presented. Boxed insets in **b** and **e** highlight the regions in **c** and **f**. Quantification of Ki67 was performed as described in the methods. Data was summarized across multiple vehicle- and 5-day or 10-day lapatinib-treated tumors and from three independent experiments. Note the overall reduction in Ki67+ cells post-lapatinib treatment (**e** and **g**). Graph (**g**) shows the percent Ki67+ cells per tumor (average per 10× field). Each line represents the mean. Graph (**g**) Mann-Whitney test vehicle versus 5-day lapatinib-treated or 10-day lapatinib-treated; *p* value < 0.0001. Note a subset of the outer niche-localized tumor cells maintain proliferative capacities post-lapatinib (**f** and **h**). Graph (**h**) shows the percent outer Ki67+ cells per tumor (average per 10× field). Each line represents the mean. Graph (**h**) Mann-Whitney test vehicle versus 5-day lapatinib-treated; *p* value = 0.0021 and vehicle versus 10-day lapatinib-treated; *p* value = 0.0707. Scale bar, ~100 μm.

**Supplementary Figure 3.** SUM190 intraductal (DCIS-like) tumors. Intraductal transplantation of SUM190 tumor cells results in the establishment of DCIS-like tumor xenografts similar to SUM225. SUM190 tumor cells, which harbor a *PIK3CA* mutation (H1047R), are insensitive to lapatinib treatment *in vivo*. H&E comparison of vehicle- (**a,b**) and lapatinib-treated (**d,e**) SUM190 DCIS-like tumors. Unlike SUM225, note no significant changes in the viable SUM190 tumor cell content. HER2 immunostains (**c,f**) highlight the HER2+ tumor cells. Scale bar, ~100 μm.

**Supplementary Figure 4.** Lapatinib induces pro-survival BCL2 in the niche-protected tumor cells. Additional vehicle- (**a** and **d**) and lapatinib-treated (**b**,**c** and **e**,**f**) tumor sections assayed for BCL2 via IHC. Note selective BCL2 induction within niche-localized tumor cells (**b**,**c** and **e**,**f**). Arrowheads (**a**-**c**) highlight regions magnified in **d**-**f**. Scale bar, ~200 μm.

**Supplementary Figure 5.** SUM225 tumors maintain an ER-negative phenotype post-lapatinib. Comparison of vehicle- (**a,b**) and lapatinib-treated (**c,d**) SUM225 tumors assayed for ERα via SP1 IHC. Arrowheads in **a** and **c** highlight the regions presented in **b** and **d**. Note BCL2 upregulation is not coupled to parallel estrogen receptor upregulation *in vivo*. Multiple tumors from two independent experiments were assayed for ER+ cells (**e**). All tumors untreated or treated were ER-negative. Scale bar, ~100 μm.

**Supplementary Figure 6.** Adverse events associated with lapatinib-based combination treatments. Mouse body weights were measured on day 0 and throughout the 15-day treatment period. Mouse weight loss at day 15 relative to day 0 is graphed as percent reduction in day 0 weight (**a**). Lapatinib plus ABT-737 associated weight loss exceeds 20% at day 15 and as such prevents combination treatment beyond this time point. Mouse blood was collected at the experimental endpoint and platelet counts were assessed by Charles River Labs (**b**). Note thrombocytopenia associated with ABT-737 treatments, confirmation of ABT-737 pharmacodynamics *in vivo* (Mann-Whitney test vehicle versus ABT-737; *p* value = 0.0002). Note the overall platelet sparing features of ABT-199 treatments (Mann-Whitney test ABT-199 versus ABT-737; *p* value = 0.0040). Each symbol represents an individual mouse. Each line represents the median. Data summarizes two independent *in vivo* experiments. Due to insufficient quantities, three blood samples were not assayed for platelet counts (one lapatinib and two lapatinib + ABT-737).

| Treatment Group | n^[[1]](#footnote-1)^ | x̄^[[2]](#footnote-2)^ | σ_x̅_^[[3]](#footnote-3)^ | N^[[4]](#footnote-4)^ |
| --- | --- | --- | --- | --- |
| vehicle | 8 | 30 | 3.0 | 241 |
| lapatinib | 8 | 25 | 4.0 | 196 |
| ABT-737 | 8 | 37 | 1.5 | 292 |
| lapatinib + ABT-737 | 8 | 37 | 2.7 | 296 |
| ABT-199 | 4 | 29 | 2.3 | 114 |
| lapatinib + ABT-199 | 5 | 25 | 5.8 | 124 |

**Supplementary Table 1.** Description of SUM225 tumors evaluated post -lapatinib and or -ABT

| Treatment Group | n^[[5]](#footnote-5)^ | x̄^[[6]](#footnote-6)^ | σ_x̅_^[[7]](#footnote-7)^ | N^[[8]](#footnote-8)^ |
| --- | --- | --- | --- | --- |
| vehicle | 5 | 29 | 0.0 | 145 |
| T-DM1 | 5 | 30 | 1.7 | 151 |

**Supplementary Table 2.** Description of SUM225 tumors evaluated post-T-DM1

**Supplementary Methods**

**Supplementary Methods Table 1.**

| **Antibody** | **Source ID** | **Species** | **Antigen Retrieval** | **Dilution** |
| --- | --- | --- | --- | --- |
| BCL-XL | CST 2764 | R | TE pH 9 Dako S2368 | 1:500 |
| BCL2 | Dako M0887 | M | TE pH 9 Dako S2368 | 1:50 |
| ERα | Dako M3634 | R | citrate pH 6 Dako S1699 or S1700 | 1:200 |
| HER2 | Dako A0485 | R | citrate pH 6 Dako S1699 or S1700 | 1:200 |
| HER2 | Epitomics 42011 | R | citrate pH6 Dako S2369 | 1:100 |
| Ki67 | Dako M7240 | M | citrate pH 6 Dako S1700 | 1:200 |
| laminin | Dako Z0097 | R | proteinase K Dako S3020 | 1:500 |
| SMA | Dako M0851 | M | TE pH 9 Dako S2367 or S2368 | 1:100 |

**Supplementary Methods**

**PFAPE or FFPE Preparation**

1. Bake slides at 60-65°C for 1h (optional). Bring reagents to room temperature (RT).
2. Deparaffinize tissue sections by xylene (two or three times, five minutes each).
3. Rehydrate tissue sections by ethanol (EtOH) series such as 100% (two times, two minutes each), 95% (two times, two minutes each), 70% (two times, two minutes each) and ddH_2_O (one time, five minutes).

**Antigen Retrieval**

1. Perform antigen retrieval as follows per primary antibody specifics.
   1. Tris-EDTA pH9 (Dako S2368), decloak chamber (40 min) plus 20 min RT
   2. proteinase K (Dako S3020), 7 min RT
   3. citrate pH6 (Dako S2369;S1700 or Sigma C9999), decloak chamber (40 min) plus 20 min RT
2. Wash slides in ddH_2_O (one time, five minutes).

**Quench**

1. Block peroxidase in 3% H_2_O_2_ (Sigma H1009) in ddH_2_O for 10 min at RT.
2. Wash slides in ddH_2_O (one time, five minutes).
3. Mark slides with a PAP pen to encircle the tissue.

**Block**

1. Block slides in a solution of 5% goat serum (Invitrogen 16210) in 1×TBST (Dako S3006) for 10 min at RT or 1 h at RT.

**Primary Antibody**

1. Prepare primary antibody in Antibody Diluent (Dako S0809 or CST8112).
2. Incubate tissue sections with primary antibody for 1 h at RT or overnight at 4°C.
3. Wash slides in 1×TBST (three times, ten minutes each).

**Secondary Antibody without amplification**

1. Incubate tissue sections with secondary antibody for 30 min at RT.
   1. anti-mouse or rabbit HRP (Dako K4000, K4002)
   2. anti-mouse or rabbit BOOST HRP (CST 8125, 8114)
2. Wash slides in 1×TBST (three times, ten minutes each).

**Secondary Antibody with amplification according to VECTASTAIN® Elite ABC Kit (PK-6100) biotinylated anti-rabbit (BA-1000) or biotinylated anti-mouse (BA-2000)**

**DAB+Counterstain**

1. Prepare sufficient DAB for batch or drop process (Sigma D4418).
2. Monitor the color development of the tissue sections.
3. Submerge slides in ddH_2_O (five minutes, RT).
4. Counterstain for 30 s - 5 min at RT in hematoxylin (Sigma MHS32).
5. Rinse slides in two changes of tap water to permit sufficient bluing (five minutes, RT).

**Mount**

1. Dehydrate slides by reverse EtOH series such as 70%, 95%, 100%, 100% EtOH (one time, 20 s).
2. Xylene (two or three times, 2-3 minutes each).
3. Mount slides with Permount (Fisher SP15) and allow slides to seal overnight.

1. n = Total number of tumor-bearing female NOD/scid mice [↑](#footnote-ref-1)
2. x̄ = Average number of DCIS-like components scored per tumor [↑](#footnote-ref-2)
3. σ_x̅_ = Standard error of the mean (x̄) [↑](#footnote-ref-3)
4. N = Total number of DCIS-like components scored for the analysis [↑](#footnote-ref-4)
5. n = Total number of tumor-bearing female NOD/scid mice [↑](#footnote-ref-5)
6. x̄ = Average number of DCIS-like components scored per tumor [↑](#footnote-ref-6)
7. σ_x̅_ = Standard error of the mean (x̄) [↑](#footnote-ref-7)
8. N = Total number of DCIS-like components scored for the analysis [↑](#footnote-ref-8)
