# Supplementary figures and images for: Niche-localized tumor cells are protected from HER2-targeted therapy via upregulation of an anti-apoptotic program in vivo
Source: NPJ Breast Cancer. 2017 May 1;3:18. doi: 10.1038/s41523-017-0020-z (PMC5460247; doi:10.1038/s41523-017-0020-z)

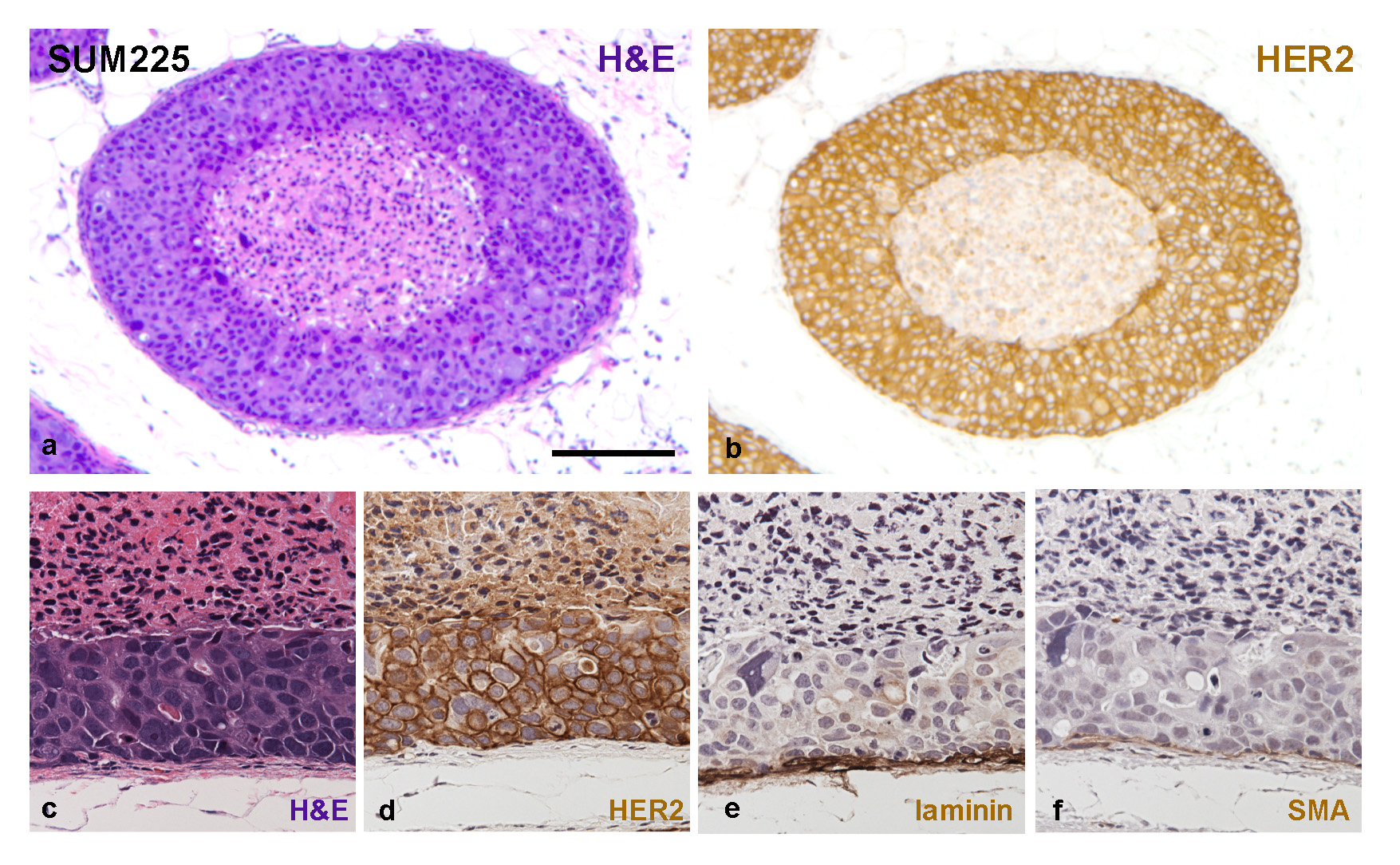

Supplement: Supplementary file 2 — Supplementary Figure 1 [file 41523_2017_20_MOESM2_ESM.jpg]

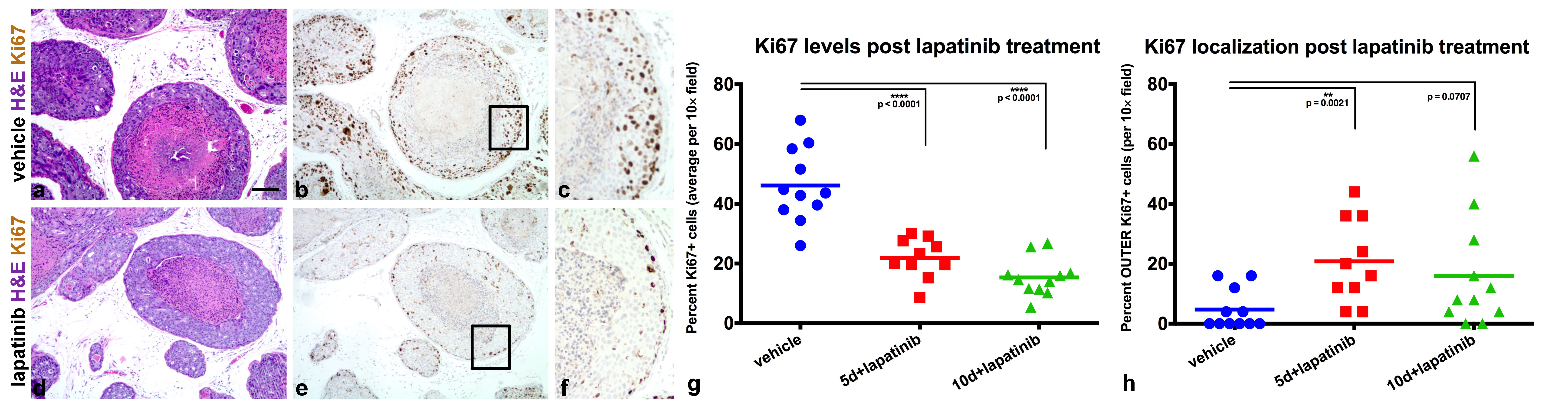

Supplement: Supplementary file 3 — Supplementary Figure 2 [file 41523_2017_20_MOESM3_ESM.jpg]

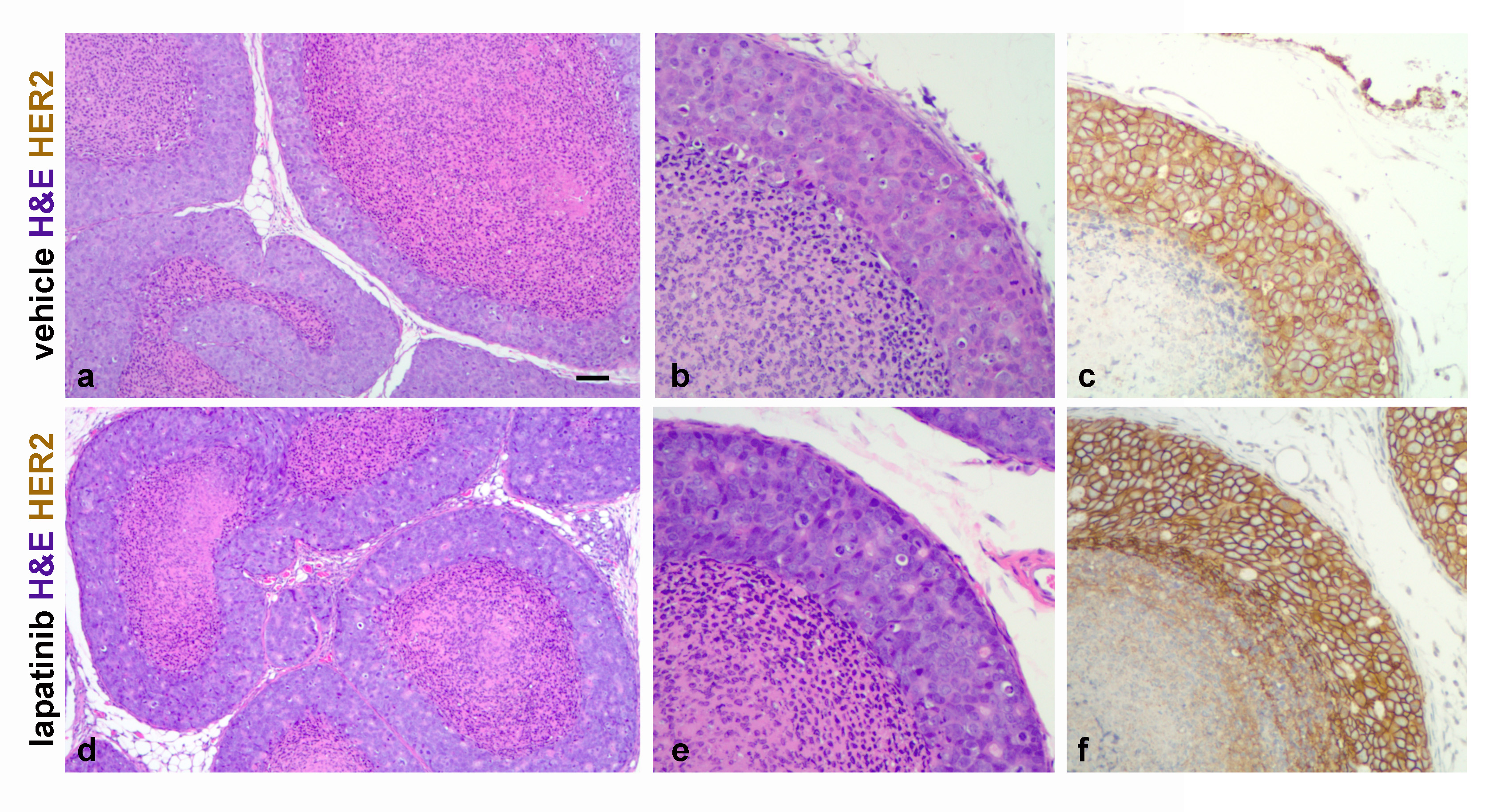

Supplement: Supplementary file 4 — Supplementary Figure 3 [file 41523_2017_20_MOESM4_ESM.jpg]

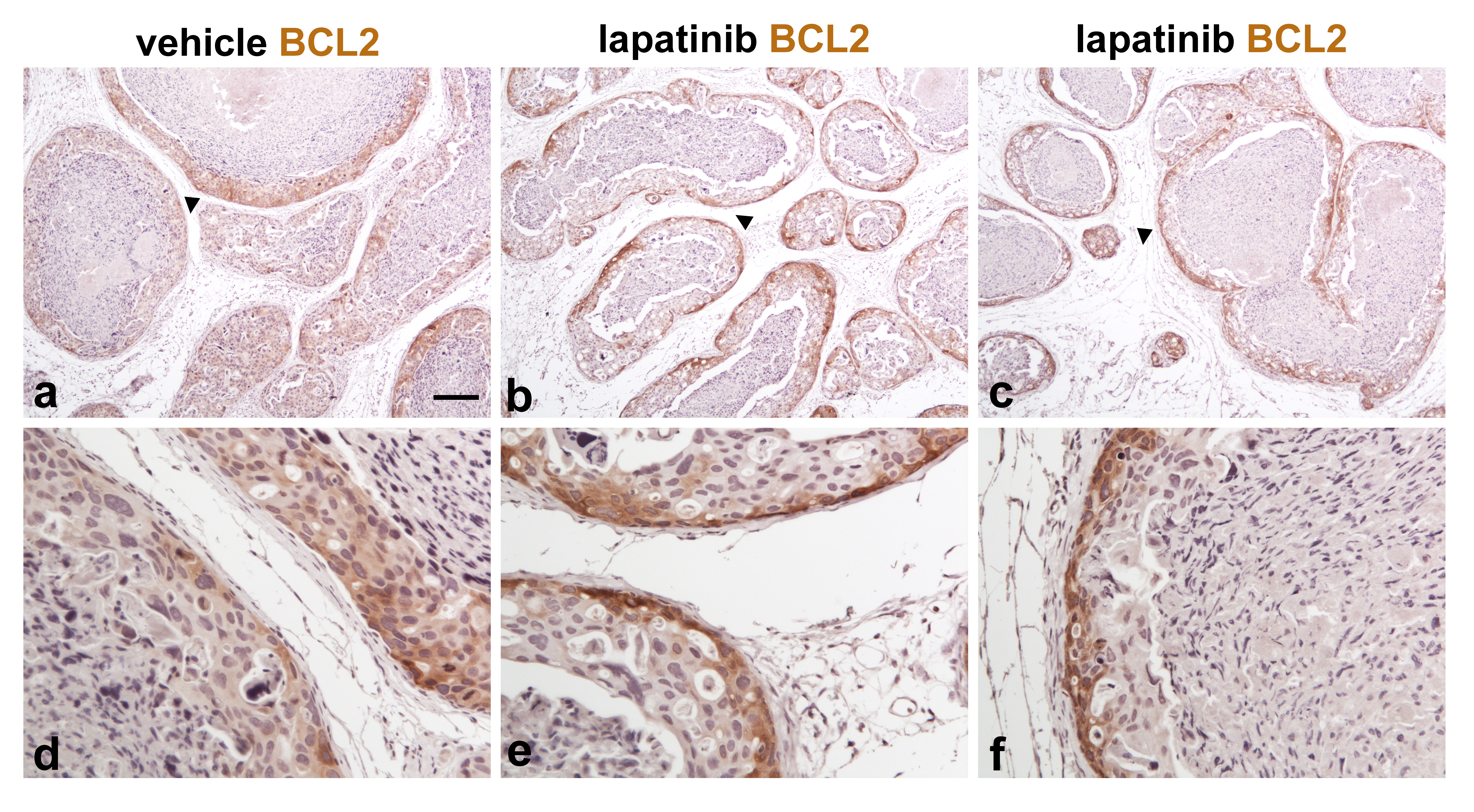

Supplement: Supplementary file 5 — Supplementary Figure 4 [file 41523_2017_20_MOESM5_ESM.jpg]

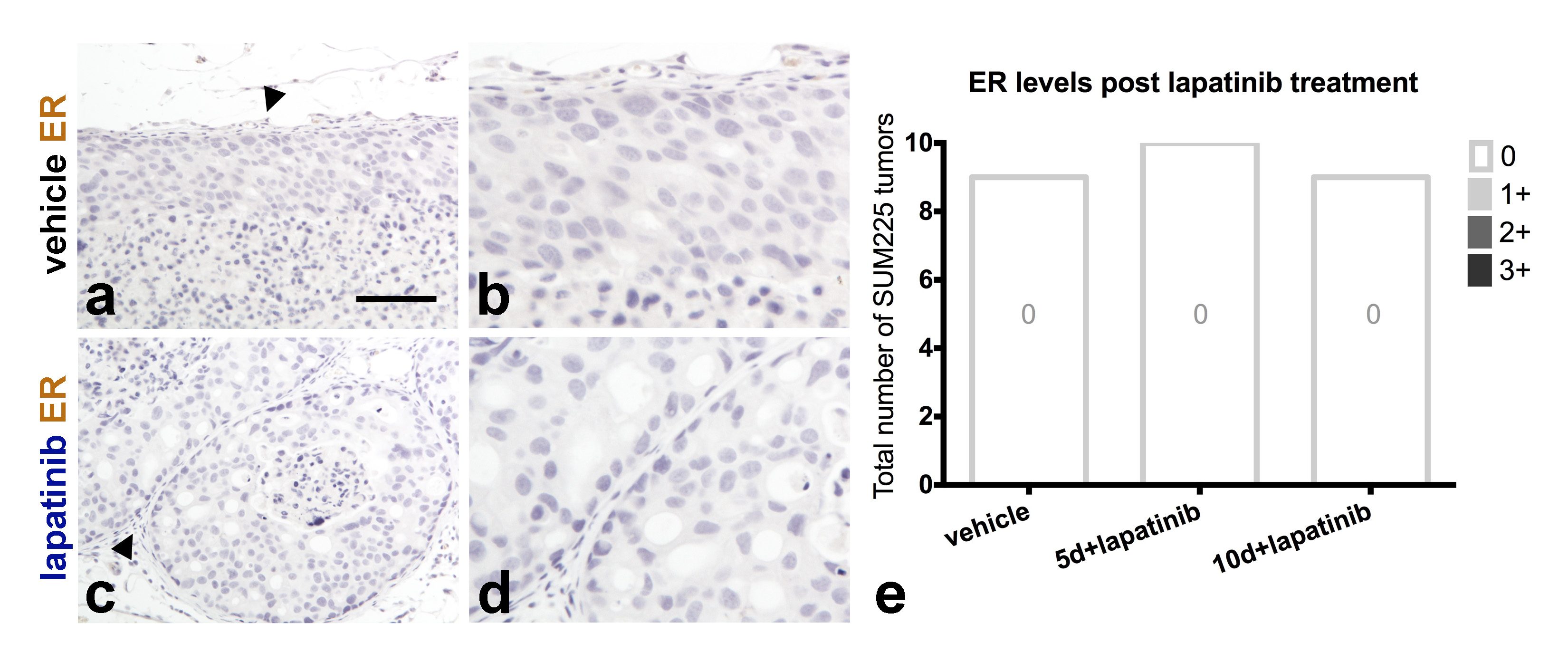

Supplement: Supplementary file 6 — Supplementary Figure 5 [file 41523_2017_20_MOESM6_ESM.jpg]

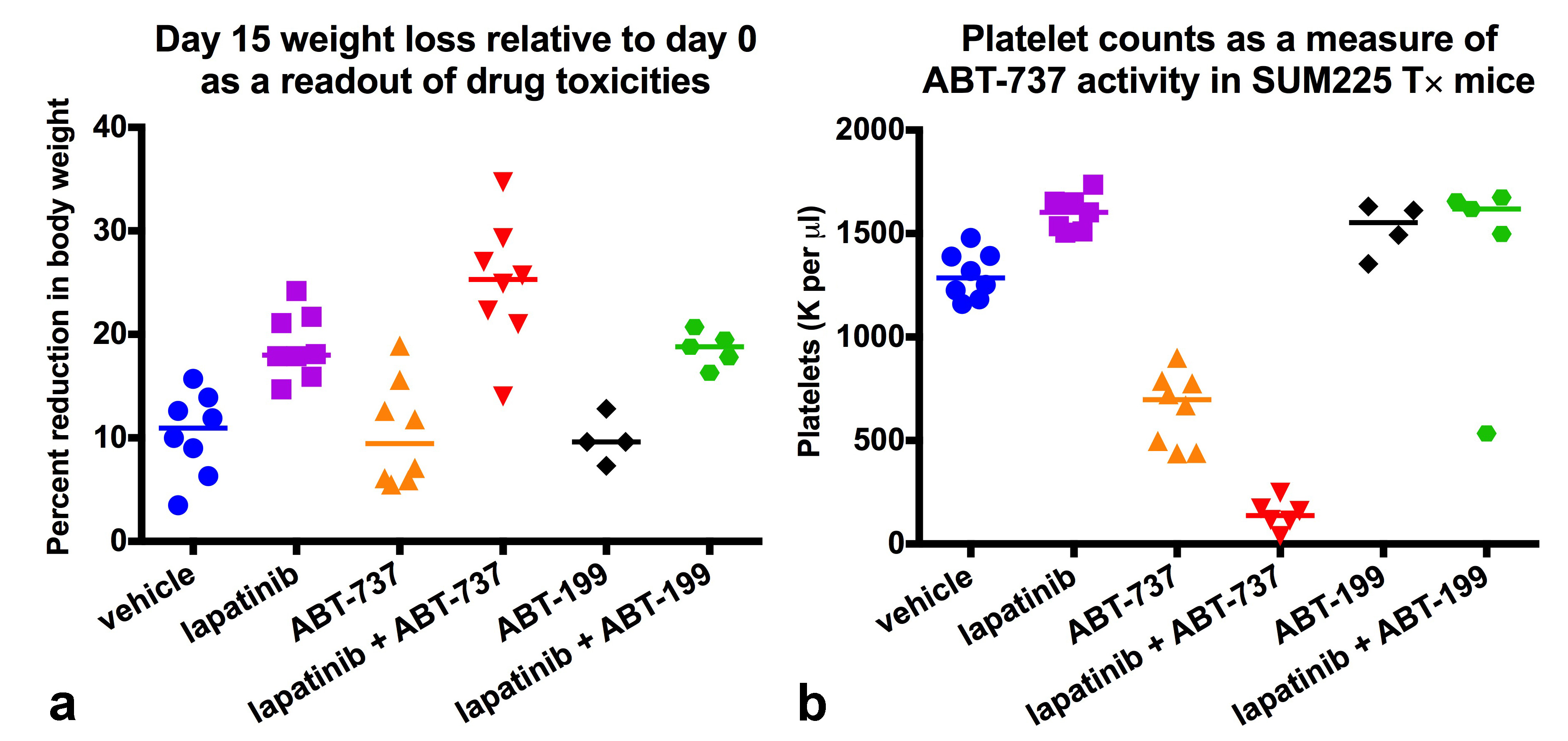

Supplement: Supplementary file 7 — Supplementary Figure 6 [file 41523_2017_20_MOESM7_ESM.jpg]
